# Veratridine, a plant-derived alkaloid, suppresses the hyperactive Rictor-mTORC2 pathway: a new targeted therapy for primary and metastatic colorectal cancer

**DOI:** 10.21203/rs.3.rs-5199838/v1

**Published:** 2024-10-25

**Authors:** Morgan M. Eikanger, Sanam Sane, Kate S. Schraufnagel, John L. Slunecka, Rashaun A. Potts, Jessica Freeling, Grigoriy Sereda, Bakhtiyor Rasulev, Reed L. Brockstein, M A Bashar Emon, M Taher A. Saif, Khosrow Rezvani

**Affiliations:** University of South Dakota Sanford School of Medicine; University of South Dakota Sanford School of Medicine; University of South Dakota Sanford School of Medicine; University of South Dakota Sanford School of Medicine; University of South Dakota Sanford School of Medicine; University of South Dakota Sanford School of Medicine; University of South Dakota College of Arts and Sciences; North Dakota State University; University of Illinois Urbana-Champaign; University of Illinois Urbana-Champaign; University of Illinois Urbana-Champaign; University of South Dakota Sanford School of Medicine

**Keywords:** Veratridine, mTORC2, Rictor, UBXN2A, metastasis, colorectal cancer, mouse

## Abstract

Despite considerable advances to improve colorectal cancer (CRC) survival over the last decade, therapeutic challenges remain due to the rapid metastatic dissemination of primary tumors and screening limitations. Meanwhile, the rise of CRC in younger adults (Early-onset CRC), commonly diagnosed with a metastatic form of the disease, shows the pressing need to develop more effective targeted therapies to decrease the high mortality rates associated with metastatic disease. Hyperactivation of the Rictor-mTORC2-AKT signaling pathway drives key metastatic players in diverse malignant tumors, including early- and late-onset colorectal cancer. Selective mTORC2 inhibitors are becoming a potential treatment strategy for CRC due to the therapeutic limitations of mTORC1 inhibitors. Veratridine (VTD), a lipid-soluble alkaloid extracted from Liliaceae plants, can transcriptionally increase UBXN2A, which induces 26S proteasomal degradation of the Rictor protein, a key member in the mTORC2 complex. Destabilization of Rictor protein by VTD decreases Akt phosphorylation on Ser^473^, which is responsible for metastatic signaling downstream of the mTORC2 pathway in diverse malignant tumors. VTD decreases the population of metastatic colon cancer stem cells and functions as an angiogenesis inhibitor. VTD effectively reduces the spheroid growth rate and restricts cell migration. Live cell migration and invasion assays alongside biomechanical-force-based experiments revealed that VTD suppresses colon cancer cell invasiveness and the ensuing risk of tumor metastasis. A CRC mouse model that mimics the natural stages of human sporadic CRC revealed that VTD treatment significantly decreases tumor growth in a UBXN2A-dependent manner. This study showed a novel mechanistic connection between a ubiquitin-like protein and mTORC2-dependent migration and invasion in CRC tumors. This study revealed the therapeutic benefit of selective inhibition of Rictor in CRC, particularly in tumors with a hyperactive Rictor-mTORC2 signaling pathway. Finally, this study opened a new platform for repurposing VTD, a supplemental anti-hypertension molecule, into an effective targeted therapy in CRC tumors.

## Background

Colorectal cancer (CRC) is the third leading cause of cancer deaths in the USA, with an estimated 53,000 deaths in 2023. Dissemination of the primary tumor to distant sites such as the liver and lungs is the major cause of death in the majority of patients, and there is a lack of therapies capable of targeting the metastatic form of the disease. There has been a significant rise in CRC incidences among individuals under 50 years old, and these early-onset patients are diagnosed in the later stages, resulting in an increased number of metastatic CRC-related deaths (1, 2). Dissemination of primary CRC tumors to distant sites such as the liver and lungs is the primary cause of death in most patients (3). With limited therapeutic options for advanced CRC, there is a clear need to develop safer and more effective targeted therapies to decrease the high mortality rates associated with metastatic disease (3, 4). The Rictor-mTORC2 signaling pathway promotes metastasis in a broad range of cancer types, including CRC (5–9). However, there are currently no active clinical trials involving selective mTORC2 inhibitors for CRC in ClinicalTrials.gov.

We previously discovered that a novel ubiquitin-like protein, UBXN2A, functions as a specific tumor suppressor protein in CRC (10–14). Consistent with a tumor suppressor role, CRC tissues with worse histological grades exhibit decreased UBXN2A protein expression (10, 15). In addition, higher UBXN2A expression correlates with improved survival probability compared to lower UBXN2A in human CRC (16). We have shown that UBXN2A can negatively regulate mTORC2 activity by selective proteasomal degradation of Rictor protein, suppressing mTORC2-Rictor’s downstream metastatic targets (5, 15). This study focuses on Veratridine (VTD), a plant alkaloid previously used as an anti-hypertensive supplement in humans (17). In a similar previously described mechanism (18), VTD elevates nuclear Zyxin, which can stimulate UBXN2A promoter activity by interacting with corresponding transcription factors (manuscript under preparation). By inducing the expression of UBXN2A in colon tissue (19), VTD can target the hyperactive Rictor-mTORC2 tumorigenic pathway in CRC cells. Therefore, VTD can emerge as a new natural-based targeted therapy for more than 50% of CRC patients with hyperactive mTORC2.

This study aimed to understand the therapeutic mechanism and efficacy of VTD as a potential UBXN2A enhancer in CRC. The profound understanding of the biological and therapeutic impact of the VTD-UBXN2A axis on the mTORC2-Rictor tumorigenic pathway at molecular, cellular, and *in vivo* levels validated VTD as a novel mTORC2 inhibitor for future preclinical and clinical research. The achieved results support the following hypothesis: the induction of UBXN2A by VTD suppresses the overdriven mTORC2 pathway using *in vitro* and *in vivo* models, resulting in suppression of migration, invasion, and stemness of CRC cells as well as the induction of apoptosis.

## Material and methods

### Spheroid culture and imaging:

Three-dimensional (3D) spheroids were generated using SpheroTribe according to the manufacturer’s instructions (IDYLLE, France). In summary, one million cells were suspended in 4mL of their respective media and 1mL of SpheroTribe. 50μL of cell dilution was added to each 96-well plate for 10,000 cells loaded per well. This protocol allows a robust generation of uniform single spheroid per well with 3D morphology characteristics that were imaged weekly before treatment using Cytation 1, a cell imaging reader with fluorescence and high-contrast brightfield imaging (Agilent, USA).

### Human apoptosis antibody array:

We used the human Apoptosis Antibody Array-Membrane (Abcam, USA, #ab134001) to detect the expression of possible apoptosis proteins enhanced by VTD. The array has 43 apoptotic markers present in intrinsic and extrinsic apoptosis pathways. The experiments and the following analysis were performed based on the manufacturer’s instructions (20). The UN-SCAN-IT gel analysis software measured the signals recorded on X-ray film using HRP-based chemiluminescence. We used two membrane apoptosis antibody array membranes per treatment, DMSO or VTD, to measure apoptotic markers in LoVo colon cancer cell line. There were two anti-body fixed spot per proteins. The arrays produced substantively similar results.

### Isolating cancer Stem cells from human colorectal cancer-derived cell lines:

To isolate individual colon cancer stem cell populations (CD44^+^ and LGR5^+^), cells were routinely cultured, harvested, and digested with Accutase (Life Technologies, USA) and resuspended in an incubation buffer (catalog: 554657, BD Biosciences, USA). 10^6^ un-permeabilized cells suspended in 100μl incubation buffer were stained with 125ng antibodies (mouse anti-human CD44-PE, catalog: 561858 or rat anti-human Lgr5-Alexa Fluor, catalog: 562903, BD Biosciences) for 30 minutes at room temperature on a gentle shaker in a dark box. After staining, cells were washed twice with incubation buffer. Washed cells were suspended in a final volume of 1 mL BD FACS Pre-Sort Buffer (catalog: 563503, BD Biosciences, USA) per sample. Sample acquisition and cell sorting were conducted on the BD FACSMelody^™^ cell sorter (BD Biosciences, USA) equipped with BD Chorus software as described previously with slight modifications (21). After sorting, cells were maintained with full-medial for downstream experiments.

### Biomechanical Assay:

The experimental setup and protocol for the assay with the biomechanical sensor have been previously described in detail (22, 23). Briefly, the sensors are prepared by pouring polydimethylsiloxane (PDMS, Sylgard 184) into a silicon (Si) mold microfabricated by photolithography. After curing at 60°C for 24 hours, the PDMS sensors are removed from the mold and placed on a sacrificial gelatin layer in a glass-bottom dish. Next, collagen solution was prepared by mixing rat tail type-I collagen (Corning) with a neutralizing solution made with 1N sodium hydroxide, 10X PBS, and deionized water (24). Collagen is then mixed with the cell suspension to have a final concentration of 2 mg/ml collagen and 2 million/ml SW480 cells preincubated in drugs for 48 (VTD 100μM) & 24 (5-FU 5μM) hours. 5μl of the cell-collagen mixture was pipetted between the grips and allowed to polymerize at room temperature for 30 mins. The tissue samples self-assemble in situ on the sensors. VTD and 5-FU treatment continued for the duration of the experiment. The setup is placed in the incubator at 37°C, allowing dissolution and washout of the sacrificial gelatin. The tissues on the sensors are monitored with time-lapse brightfield imaging to determine force dynamics. The force and stiffness of the tissues were measured by applying compression and tension with a 3D linear stage.

### xCELLigence real-time cell analysis to measure cell migration and invasion:

Cell migration and invasion assessments were performed in real-time using xCELLigence technology, as previously described (15). Briefly, HCT-116 and HT-29 cells were treated with vehicle control (DMSO) or VTD (100μM) for 72 hours before starting migration and invasion experiments. For migration, 100μL of media containing 40,000 HCT-116 and 60,000 HT-29 cells was loaded into the upper chamber of the CIM-Plate. For invasion, 20μL of Corning Matrigel Matrix (Catalog number#CB-40234, USA) was loaded into the upper chamber of the CIM-Plate and allowed to polymerize. Then, 100μL of media containing the designated number of cells was added to the upper chamber. The xCELLigence system measured the impedance value of each well every 15 minutes for 120 hours. The collected raw data is presented as a CI value for each experiment. The critical time point for migration was between 40 and 90 hours. The critical time point for the invasion was between 30 to 50 hours. GraphPad Prism 10 was used to analyze the cell migration and invasion rate, as the slope (1/hr), during the critical time points for HCT-116 and HT-29 cells.

## RESULTS

### Veratridine targets the Rictor protein in human colon cancer.

**Figure 1:**
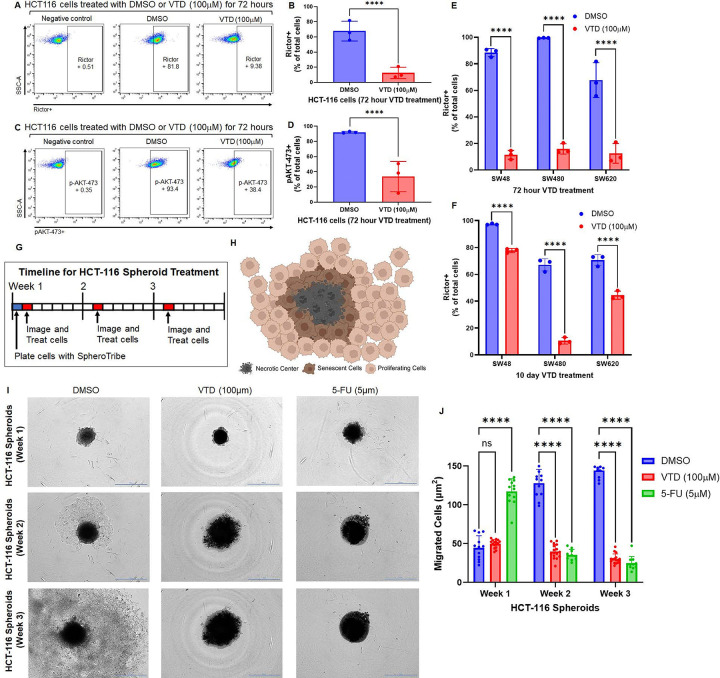
VTD significantly decreases Rictor and p-AKT^473^ levels in human colon cancer cells. HCT-116 colon cancer cell lines were treated with DMSO or VTD (100μM) for 72 hours and were stained with anti-Rictor (APC) (**A-B**) or anti-p-AKT^473^ (Alexa Fluor 647) (**C-D**) for flow cytometry analysis. There is a significant reduction of Rictor and its downstream protein, p-AKT^473^, in SW48, SW480, and SW620 primary and metastatic CRC cells treated 72 hours (**E**) and 10 days (**F**) with VTD (n=3, ****p<0.0001, mean±SD). In another set of experiments, 24 hours after the establishment of HCT-116 spheroid cells, the 3D cells were treated with DMSO, VTD (100μM), and 5-FU (5μM). Spheroids were treated weekly and imaged using Cytation 1 (**G**). Spheroids are comprised of necrotic, senescent, and proliferating cells (**H**). Spheroid migration proliferating cells was determined using by ImageJ software by measuring the area of the necrotic core and subtracting it from the total spheroid area (necrotic core + senescent + proliferating cells). VTD and 5-FU treated cells had significantly less migration starting after two weeks of treatment (**I-J**). (n≥10, ****P<0.0001, mean ± SD).

We hypothesized that UBXN2A induced by VTD can reduce the level of Rictor protein. HCT-116 colon cancer cells were treated with DMSO or VTD (100μM) for 72 hours. We have shown that 100μM of VTD can significantly increase the RNA (19) and protein level of UBXN2A in diverse CRC cancer cell lines (Fig. S1) and animal models (10, 14). 72-hour VTD (100μM) treatment significantly decreased the percentage of Rictor-positive cells, showing that VTD could reduce Rictor in HCT-116 (Fig. 1A–B). To determine if the downregulation of Rictor in VTD-treated cells has a biological impact downstream of the mTORC2 signaling pathway, we chose to examine the phosphorylation level of AKT^473^, the direct target of activated Rictor in cancer cells. (25). VTD (100μM) treatment in HCT-116 cells downregulates the phosphorylation level of AKT^473^, an essential step for the AKT-mediated phosphorylation of different downstream signaling pathways important in tumorigenesis, including tumor cell migration, angiogenesis, and metastatic colon cancer stem cells (Fig. 1C–D) (26).

In the next set of experiments, we determined the status of Rictor-p-AKT^473^ in SW48, SW480, and SW620 colon cancer cell lines. SW480 and SW620 colon carcinoma cell lines are derived from primary and secondary tumors resected from the same patient, providing a valuable resource for evaluating the status of Rictor and p-AKT^473^ during the stage transition of cells from primary to metastatic in the presence of VTD (27). Acute VTD (100μM) treatment (72 hours) significantly decreased the level of Rictor in all three increasingly metastatic CRC cell lines (Fig. 1E, Fig. S2). To evaluate if cancer cells can develop resistance toward the negative regulatory effect of VTD on the Rictor-p-AKT^473^ pathway, we used a sub-chronic (10-day) treatment. After 10-day VTD (100μM) treatment, Rictor protein still significantly decreased in all three cell lines, especially SW480 cells (Fig. 1F and Fig. S3). Expectedly, SW620 cells showed a greater percentage of cells positive for Rictor after ten days compared to the 72-hour treatment, which could be attributed to the fact that metastatic cells can promote drug resistance faster than non-metastatic cells. While SW480 and SW620 carry identical mutation profiles, these cells have epigenetic differences. The development of drug resistance in SW48 in response to sub-chronic VTD is possibly due to its hyperactive mutation in EGFR, which contributes to drug resistance in diverse tumors (28). VTD (100μM) treatment significantly reduced p-AKT^473^ in all three cell lines (Fig. S4).

Next, we examined the anti-growth and anti-migratory function of VTD on HCT-116 tumor spheroids (Fig. 1G). This three-dimensional (3D) system can mimic drug resistance developed in tumors with enrichment CSCs or cells with stem cell-related characteristics allowing for a physiological approach. We used 5-Fluorouracil (5-FU) as the positive control because it is the first-line chemotherapy drug used in CRC patients and it inhibits migration and invasion of CRC cells through the PI3K/AKT pathway (29). Migrating cells, including the senescent and proliferating cells outside of the necrotic center, were measured as previously described (Fig. 1H and Fig. S5) (30, 31). VTD (100μM) and 5-FU (5μM) could significantly reduce the area of migrated cells and proliferation rate of cells compared to DMSO after three weeks (Fig. 1I–J). VTD (100μM) inhibits cancer cell migration and enriches non-spheroid integrating cells as effectively as 5-FU (5μM) (Fig. 1I–J). 5-FU treated cells have the most migrating cells in the first week, which could be attributed to the fact that 5-FU can activate and enrich cancer stem cells (CSCs) in the early phase of treatment, which is overcome by continuing treatment (Fig. S6) (32). These results indicate that the VTD-UBXN2A axis can effectively inhibit the mTORC2 signaling pathway, which is mediated through a fully phosphorylated AKT signaling cascade.

### Veratridine decreases cancer stem cell populations

**Figure 2:**
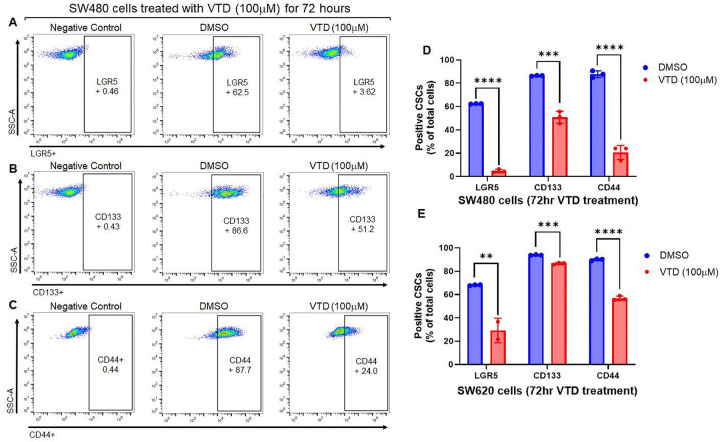
VTD significantly decreases colon cancer stem cell populations in primary colon cancer cells. Enrichment of positive CSCs, such as Lgr5, CD44, and CD133 populations, are supported by the mTORC2 signaling pathway. SW480 (primary, **A-D**) and SW620 (metastatic, **E**) cells were treated with VTD (100μM) for 72 hours and stained with anti-CD44 (PE), anti-CD133 (APC), and anti-Lgr5 (Alexa Fluor 647). Individually positive cells for CSC markers were isolated by flow cytometry and analyzed using FlowJo. Expectedly, metastatic cells (SW620) revealed more resistance to VTD compared to SW480. (n=3 in triplicate, *P<0.05, **P<0.01, ***P<0.001, ****P<0.0001, mean ± SD).

Colon cancer stem cells (CSCs) are responsible for tumor metastasis and recurrence due to their tumorigenic and aggressive features, resulting in drug resistance and poor survival (33). The hyperactive mTORC2-AKT signaling pathway increases the population of CRC Colon cancer stem cells (CSCs), which induces CRC migration and enhances the metastatic dormancy of colorectal cancer (34). UBXN2A suppresses the stemness of colon cancer cells dominantly through the Rictor-mTORC2 pathway (15). We hypothesized that elevated UBXN2A by VTD decreases CSC populations. We used the following three prognostic CSC markers that dominantly contribute to the progression and maintenance of tumor migration and metastasis in CRC tumors: Lgr5^+^, CD44^+^, and CD133^+^ (35). Flow cytometry analyses showed the status of these markers in SW480 and SW620 cells after 72-hour VTD (100μM) treatment. The results revealed a higher sensitivity of Lgr5^+^ (Fig. 2A, D), CD133^+^ (Fig. 2B, D), and CD44^+^ (Fig. 2C–D) CSCs to VTD in the primary SW480 cell line versus its lymph node metastatic variant SW620 (Fig. 2E and Fig. S7). Enriched CSC populations and multiple dysregulated signaling pathways (36) allow metastatic SW620 cells to demonstrate a higher level of drug resistance. It has been well accepted that various tumorigenic pathways, such as Wingless/Integrated (WNT), Hedgehog, Notch, and PI3K/AKT/mTOR pathways, are associated with diverse CSC populations. Therefore, the different sensitivities of the three populations of CSCs to VTD within each cell line further support the selectivity of the VTD-UBXN2A axis toward the mTORC2 pathway and not to other dysregulated pathways. Several small-molecule inhibitors of selective tumorigenic signaling pathways, such as sonidegib, a potent inhibitor of Hedgehog signal transduction, have become FDA-approved CSC inhibitors in the clinic. The reduction of stemness-related markers in the presence of VTD suggests that VTD can potentially develop into a single or combination therapy to inhibit CSC populations.

### VTD effectively targets sorted CD44 + and LgR5 + positive stem cells, which strictly defines cancer stem cells in CRC.

**Figure 3:**
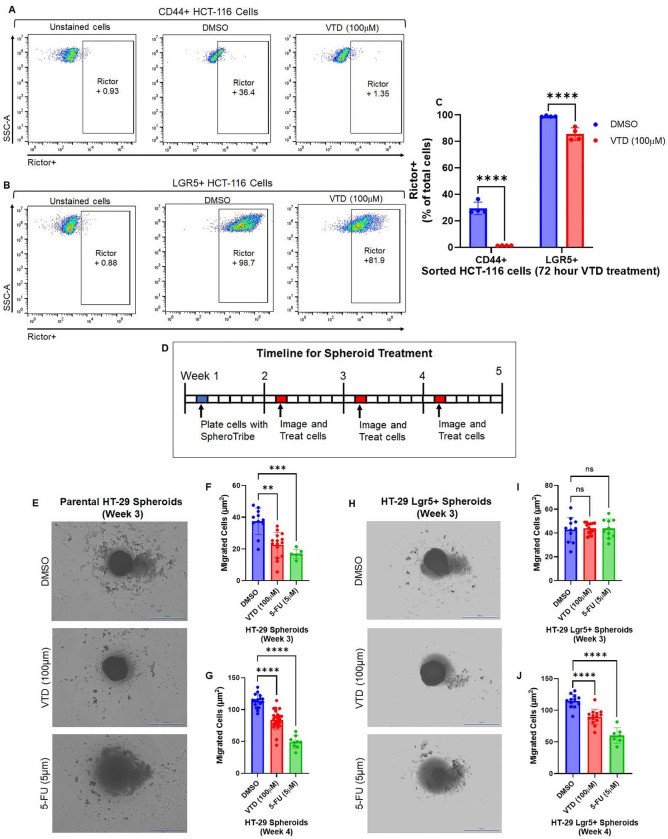
VTD dominantly downregulates Rictor protein in sorted CD44- and Lgr5-positive colon cancer stem cells. The populations of CD44 and Lgr5 CSCs are considered key prognostic factors in patients with CRC. The wild-type parental HCT116 cells were stained with anti-CD44 (PE) or anti-Lgr5 (Alexa Fluor 647) and were isolated using BD FACSMelody cell sorter technology (Fig. S8A). Significantly enriched CD44+ and LGR5^+^ CSCs were re-plated, and after three passages, cells were treated with DMSO or VTD (100μM) for 72 hours. Cells were then stained with anti-Rictor (APC) and subjected to flow cytometry an analyzed using FlowJo. VTD (100M) significantly decreased Rictor in both sorted CD44^+,^ or Lgr5^+^ CSCs (**A,C**). Interestingly, the downregulation effect of VTD on the protein level of Rictor was moderate in LGR5+ CSCs compared to CD44^+^ (**B-C**) (n=3 in triplicate, ****P<0.0001, mean ± SD). To further confirm VTD’s inhibitory effect on the Rictor-mTORC2 pathway consequently leads to the inhibition of CSCs’ migratory ability, we used SpheroTribe to generate parental HT-29 and HT-29 Lgr5+ spheroids. The HT-29 LGR5+ with mutant p53 represents a highly resistant form of CRC. HT-29 parental cells were stained with anti-Lgr5+ and sorted as previously described before spheroid generation. Treatment with DMSO, VTD (100μM), and 5-FU (5μM) began one week after spheroid generation (**D**). Parental HT-29 spheroids treated with VTD and 5-FU had significantly less migration after just one week of treatment, week 3, (**E-F**), and even less after two weeks, week 4 (**G**). Sorted LGR5+ HT-29 spheroids showed no significant response to VTD and 5-FU at week 3 (**H-I**). However, at week 4, after two treatments, VTD and 5-FU-treated HT-29 Lgr5+ spheroids had less migration compared to DMSO-treated spheroids (**J**). (n≥10, **P<0.01, ***P<0.001, ****P<0.0001, mean ± SD).

Previous biological studies, followed by a meta-analysis, demonstrated that elevated CD44-positive colon cancer stem cells are an unfavorable prognostic factor in patients with CRC. The CD44 population is responsible for poor differentiation, lymph node metastasis, and distant metastasis (37). We used cell sorting technology to isolate CD44-positive colon cancer cells from HCT-116 parental cells (Fig. S8A-D). CD44 + cells were re-plated separately from HCT-116 parental before treatment. Treatment with VTD (100μM) led to a significant reduction in the population of CD44 + cells (Fig. 3A,C). Using the same sorting approach, we isolated LGR5-positive stem cells from HCT-116 parental cells (Fig. S8E-F). The expression of Lgr5 directly correlates to the malignant biological behavior of CRC, as Lgr5 + cells promote local and distant metastases in CRC patients (38). The LGR5 + population cells show a significant but moderate response to the VTD (100μM) (Fig. 3B–C). The low sensitivity of LGR5 + cells to VTD can be due to their plasticity and ability to convert to other stem cells, which are observed with other anti-cancer drugs in CRC (39). HCT-116 cells with wild-type p53 are more sensitive to current treatment regimens than HT-29 cells with mutant p53 (40). Meanwhile, the 3D spheroid promotes the population of stem cells, which influences cell proliferation, survival, and migration. Treating spheroids derived from parental HT-29 cells compared to sorted HT-29 Lgr5 + cells allow us to validate the therapeutic efficacy of VTD on CSCs enriched in a 3D multicellular model. HT-29 parental cells were stained with anti-LGR5 and were isolated using BD FACSMelody before treatment. Parental HT-29 and HT-29 Lgr5 + spheroids began receiving VTD (100μM) or 5-FU (5μM) treatment one week after establishing their initial 3D cellular structures (Fig. 3D). HT-29 spheroids treated with VTD (100μM) and 5-FU (5μM) had significantly less proliferation and migration than DMSO-treated spheroids at weeks 3 and 4 (Fig. 3E–G, Fig. S9A). On the other hand, HT-29 CSCs Lgr5 + spheroids showed no significant response to VTD at week 3 (Fig. 3H–I). However, the proliferation and migration of HT-29 Lgr5 + spheroids significantly responded to VTD at Week 4 (Fig. 3H, J, and Fig. S9B). The spherical shape of the HT-29 became more irregular or non-spheroids, particularly in VTD and 5-FU treated groups, in a time-dependent manner. As previously described (41), the disruption and structural changes to the spheroids in the presence of 5-FU further validate VTD’s antitumor activity and its capability of targeting CSC populations.

### VTD induces apoptosis via intrinsic and extrinsic pathways.

**Figure 4:**
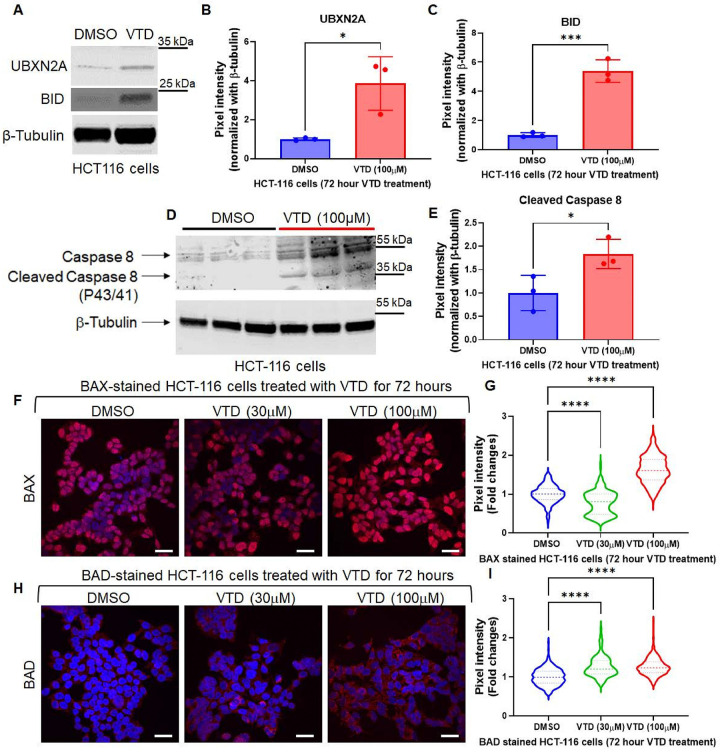
VTD induces intrinsic and extrinsic apoptotic pathways in colon cancer cells. The human antibody apoptosis array revealed that VTD induces both intrinsic and extrinsic apoptotic pathways in the LoVo colon cancer cell line (Fig. S10C). HCT-116 cells treated with VTD (100μM) had enhanced expression of UBXN2A which led to the elevation of BID protein, a pro-apoptotic member of the Bcl-2 protein family (**A-C**). VTD (100μM) significantly elevated the level of caspase 8 and its active form, cleaved caspase 8, an initiator of the extrinsic apoptotic pathway (**D-E**). Immunofluorescence experiments revealed that HCT-116 cells treated with VTD (100μM) for 72 hours upregulated BAX (**F-G**) and BAD (**H-I**), two apoptosis markers, in a dose-dependent manner. (n=3 in triplicate, *<0.05, ***P<0.001, ****P<0.0001, mean ± SD).

Drug-induced apoptosis suppresses the proliferative cells in the spheroid by inducing apoptosis (42). Therefore, we decided to understand the apoptotic mechanism of VTD targeting cancer cells. Anti-cancer molecules induce intrinsic and extrinsic apoptotic pathways to suppress tumor growth effectively. Targeting the extrinsic apoptotic pathway, which triggers cell death independent of the p53 tumor suppressor protein, provides a unique therapeutic strategy to induce apoptosis in cancer cells. Therefore, we decided to determine whether VTD targets the extrinsic pathway alongside the intrinsic pathway (43, 44). We have shown that VTD induces intrinsic apoptotic pathways in diverse colon cancer cells with and without WT-p53 (10). We treated LoVo, an APC/RAS mutant colon adenocarcinoma cell line, with VTD (10μM and 100μM). for 48 hours significantly increased Annexin V, an apoptotic marker, in a dose-dependent manner (Fig. S10A-B). Using a human apoptosis antibody array, which simultaneously detects 43 human apoptotic proteins involved in intrinsic and extrinsic pathways, we examined the status of Lovo cells’ extrinsic and intrinsic pathways treated with DMSO and VTD (100μM) (Fig. S10C). Equal total lysate protein collected from DMSO and VTD-treated LoVo cells were loaded on the Abcam Apoptosis Antibody Array membrane and exposed to X-ray films. We found an elevation greater than or equal to 25% in 32 out of 43 apoptotic proteins involved in both apoptotic pathways in VTD-treated cells versus control groups (Fig. S10C). We observed about 40% elevation of Caspase 8 and BID proteins in VTD-treated cells (Fig. S11A-C), two major components of the extrinsic pathway (40), indicating that VTD can activate both extrinsic and intrinsic pathways. Western blot analysis revealed that HCT-116 cells treated with VTD (100μM) for 72 hours elevated UBXN2A and led to a significant increase in BID protein (Fig. 4A–C) as well as the activated form of Caspase 8 (cleaved caspase 8) (Fig. 4D–E). Next, we determined the status of two apoptotic markers downstream of BID, BAX and BAD. It has been shown that the activation of BID leads to the oligomerization of BAX, which results in the release of cytochrome c (45). BAD is an important factor in activating the BAK/BAX signal (46). Additionally, activation of the PI3K/AKT pathway can control apoptosis by inhibiting BAD and BAX, slowing apoptosis (47). HCT-116 cells treated with DMSO or VTD (30μM and 100μM) were stained for BAX (Fig. 4F–G) and BAD (Fig. 4H–I). Activation of BID by VTD leads to the elevation of BAX and BAD, which are necessary for the induction and progression of the apoptotic cascade. We determined SW48, SW480, and SW620 cell viability in our sub-chronic VTD treatment based on the apoptosis results. A crystal violet assay revealed that sub-chronic treatment of colon cancer cells with VTD (100μM) can significantly decrease and, more importantly, maintain low cell viability regardless of the primary or metastatic stages of cells (Fig. S12).

Altogether, these results indicate that VTD may have a dual function in apoptosis in cancer cells. VTD activates the intrinsic and extrinsic apoptotic pathways in a UBXN2A-dependent and likely in a UBXN2A-independent manner. Simultaneously, the VTD-UBXN2A axis suppresses the inhibitory effect of the PI3K/AKT pathway on the apoptotic pathway. This dual targeting function of both the apoptotic and AKT pathways is mediated through the UBXN2A-Rictor axis, generating a progressive apoptotic cascade in cancer cells (Fig. S13D). Our ongoing study will evaluate each protein elevated by VTD shown in the apoptotic array to determine whether the VTD-UBXN2A axis and its interaction with the Rictor-mTORC2-pAKT pathway, has a novel inhibitory function on apoptosis, preferentially in human CRC.

### Inhibition of VEGF signaling by VTD through Rictor-mTORC2-pAKT inhibition

**Figure 5:**
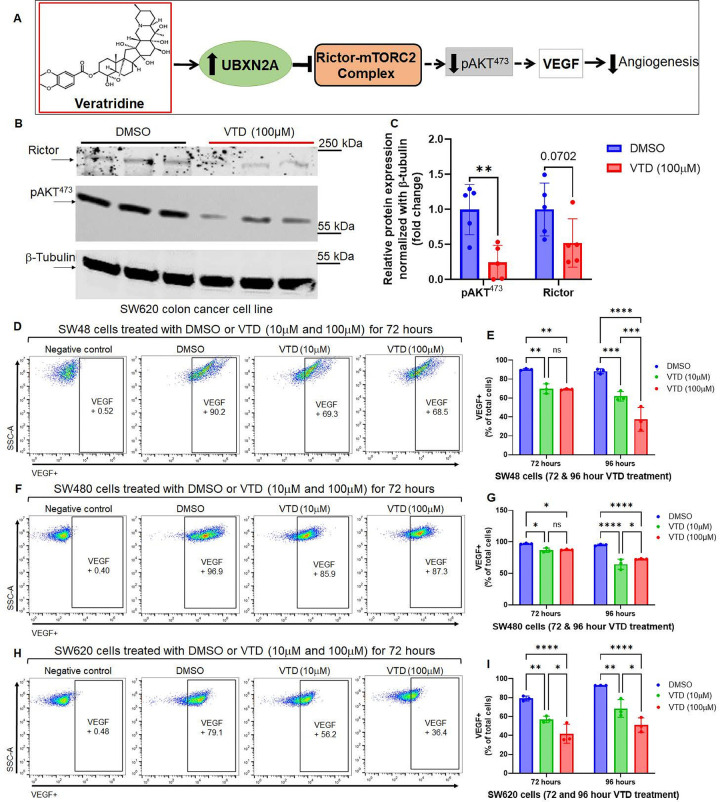
Suppression of the Rictor-mTORC2-pAKT^473^ signaling by VTD downregulates VEGF, a key inducer of metastatic angiogenesis (**A**). Rictor and p-AKT^473^ protein levels in SW620 metastatic CRC cells decreased after 72 hour VTD (100μM) treatment (**B-C**). Rictor and its downstream target, p-AKT^473^ support the formation and maintenance of angiogenesis in progressing tumors. A set of flow cytometry experiments in SW48, SW480, and SW620 cells treated with DMSO or VTD (10μM and 100μM) for 72 hours revealed that VTD moderately but significantly downregulated the level of VEGF, even at a lower dose of 10μM (**D-I**). The experiment was repeated but cells were treated for 96 hours to further provide evidence of the moderate influence of VTD on angiogenesis (**E,G,I** and Fig. S15). The moderate inhibitory effect of VTD on VEGF can beneficially decrease the development of drug resistance generated by the current potent VEGF inhibitor in the clinic. (n=3, *p<0.05, **p<0.01, ***p<0.001, ****P<0.0001, mean ± SD).

The PI3K-mTORC2 axis is central in activating VEGF-regulated phosphorylation, which facilitates metastasis and results in a poor prognostic factor in CRC survival (48). Selective mTORC2 inactivation does not allow a rapid progression of tumor angiogenesis (49, 50). Our results indicate that UBXN2A overexpression can relatively decrease the level of VEGF in HCT116 and LoVo cells (Fig. S14). Therefore, we hypothesized that the suppression of the mTORC2 pathway by VTD leads to a reduction in p-AKT^473^, resulting in a decrease of VEGF in colon cancer cells in a VTD-UBXN2A manner (Fig. 5A). Western blot experiments confirmed that VTD (100μM) can decrease the level of Rictor in SW620 metastatic cells, resulting in a significant reduction of p-AKT^473^ (Fig. 5B–C). The inhibition of angiogenesis has introduced a perplexing dilemma since the VEGF inhibitors can not only successfully reduce primary tumor growth but also promote tumor invasiveness and metastasis. Complete shutdown of angiogenesis can initiate mechanisms that increase metastatic features of tumors, such as epithelial-mesenchymal transition (EMT) (51, 52). Therefore, it has been suggested that moderate inhibition of angiogenesis rather than completely depriving tumors of vascular support can slow the development of metastatic characteristics and early-drug resistance while delivering a therapeutically significant amount of drug to the tumor mass. Therefore, we conducted a set of flow cytometry experiments to determine the status of VEGF in SW48, SW480, and SW620 cell lines after VTD treatment (10μM and 100μM) for 72 and 96 hours (Fig. 5D,F,H, and Fig. S15). Interestingly, bivariate plots (Fig. 5E, G, I) indicate that VTD decreases VEGF levels at low and high dosages at both time points. However, the percentage of inhibition was moderate (10–50%) in a cell-dependent manner. The moderate anti-VEGF function of VTD can potentially block primary tumor growth while at the same time avoiding pro-metastatic effects generated by potent VEGF inhibitors.

### Veratridine suppresses migration and invasion in human colon cancer cells.

**Figure 6:**
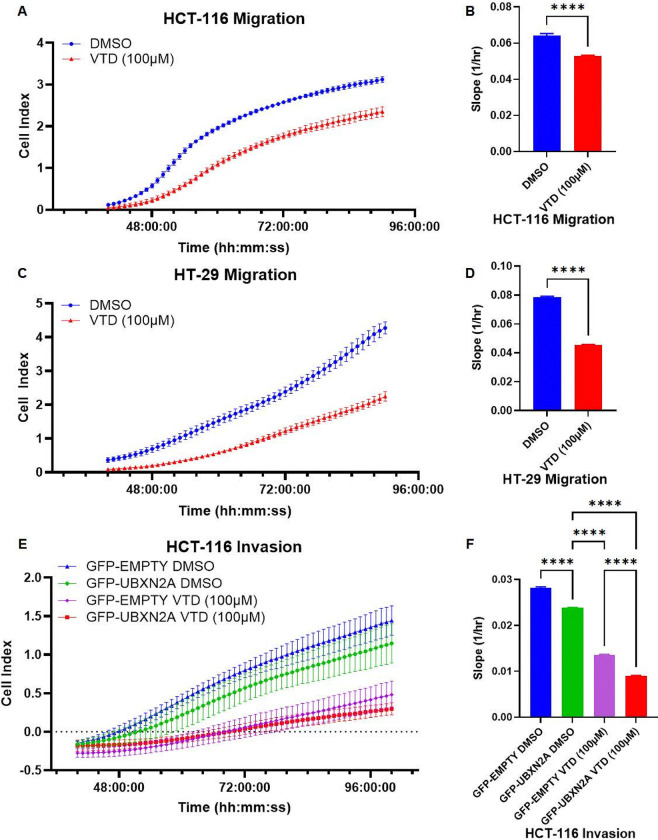
VTD suppresses colon cancer migration and invasion in a UBXN2A-dependent manner. HCT-116 (WT-p53) (**A-B**) and HT-29 (mutant-p53) (**C-D**) cells were treated with DMSO or VTD (100μM) for 72 hours before they were plated into 16-well CIM plates for cell migration using xCELLigence Real-Time technology (RTCA). Treatment continued over the duration of the experiment. Defined critical time points (~35–50 hours), recommended by the RTCA Software Pro, were used to calculate the slope per treated cell. The calculated slope of 3 separate experiments with an n of 4 wells per treatment revealed significant inhibition of cell migration in the presence of VTD regardless of the status of the p53 tumor suppressor protein (**B, D**). Next, HCT-116 cells stably expressing GFP-empty or GFP-UBXN2A (Tet-on expression system) were pre-treated with DMSO or VTD (100μM) for 72 hours before they were plated into 16-well CIM plates. Treatment continued over the duration of the experiment and cell invasion was monitored by RTCA (**E-F**). Exogenous elevation of UBXN2A by GFP-UBXN2A (**F**, green) had significant decreased invastion compared to GFP-empty (**F**, blue), showing that elevated UBXN2A can reduce invasion. Remarkably, endogenous elevation of UBXN2A by VTD (**F**, purple) had even less invasion than exogenous elevation of UBXN2A alone (**F**, green), showing VTD’s negative effect on invasion is more potent than induced exogenous UBXN2A. Upregulation of exogenous (GFP-UBXN2A) and endogenous (VTD, 100μM) UBXN2A had the least invasion (F, red). The synergistic inhibitory effect of endogenous and exogenous UBXN2A on cancer invasion indicates that VTD’s anti-migration and invasion effects is largely mediated via UBXN2A. (n=4 per experiment, ****P<0.0001, mean ± SD).

Rictor-mTORC2 pathway contributes to migration and invasion, and it has been reported that disruption of this pathway suppresses cell adhesion and migration (53–55). We have shown that elevated UBXN2A can suppress colon cancer cell migration (15). We hypothesized that elevated UBXN2A in the presence of VTD suppresses colon cancer migration and invasion. We used xCELLigence Real-Time Cell Analysis (RTCA) technology to determine the migration of HCT-116 (WT-p53) and HT-29 (mutant p53) colon cancer cells. HCT-116 and HT-29 utilize the overactivated Rictor-mTORC2 tumorigenic pathway which facilitates the development of migration and invasion (15, 56). Cells were treated with DMSO or VTD (100μM) and loaded into 16-well CIM plates that contain gold biosensors embedded in the bottom of each well that record the cells in real-time by the xCELLigence system (57). The migration rate was monitored live as previously described by our group (15). HCT-116 (Fig. 6A–B) and HT-29 (Fig. 6C–D and Fig. S16) demonstrate that VTD can significantly suppress colon cancer cell migration regardless of p53 tumor suppressor protein status. These results suggest that the VTD-UBXN2A axis dominantly decreases cancer cell migration downstream of the mTORC2 pathway. We completed invasion assays to justify further that VTD functions as a potent anti-cancer molecule by targeting the overactivated mTORC2 pathway by the UBXN2A-Rictor axis.

HCT-116 cells stably expressing GFP-empty or GFP-UBXN2A (15) were loaded into 16-well CIM plates containing Corning Matrigel matrix. For this invasion assay, cells had to invade through a layer of Matrigel matrix before they were able to stimulate the electrode sensor (57). DMSO-treated HCT-116 GFP-UBXN2A (Fig. 6E–F, green) had significantly less invasion than DMSO-treated HCT-116 GFP-Empty (Fig. 6E–F, blue) cells. More importantly, HCT-116 GFP-Empty cells treated with VTD (100 μM) (Fig. 6E–F, purple) had significantly less invasion than the exogenous elevation of UBXN2A by GFP-UBXN2A alone (Fig. 6E–F, green). VTD (100μM) treated HCT-116 GFP-UBXN2A cells had the slowest invasion of all treatment groups (Fig. 6E–F, red). This data revealed that the co-elevation of endogenous UBXN2A by VTD and exogenous UBXN2A by GFP-UBXN2A generates a dominant suppression of HCT-116 cell invasion. These results suggest the suppressory function of VTD on tumor migration and invasion is dominantly mediated through UBXN2A.

### Alteration of mechanobiological properties of colon cancer cells by VTD halts migration and invasion.

Drugs targeting mechanobiology are an emergent paradigm in cancer therapeutics. Increased tumor stiffness and high contractility facilitate metastasis by regulating the chemo-mechanical crosstalk between cancer and stromal cells (58–64). It has been shown that Rictor-mTORC2 has a significant biological impact on matrix remodeling by targeting members of matrix metalloproteinase proteins (MMP-2 and MMP-9) (65–67). Upregulation of matrix MMP-2 and MMP-9 in carcinomas allows colon cancer cells to gain the ability to invade and metastasize (68).

**Figure 7:**
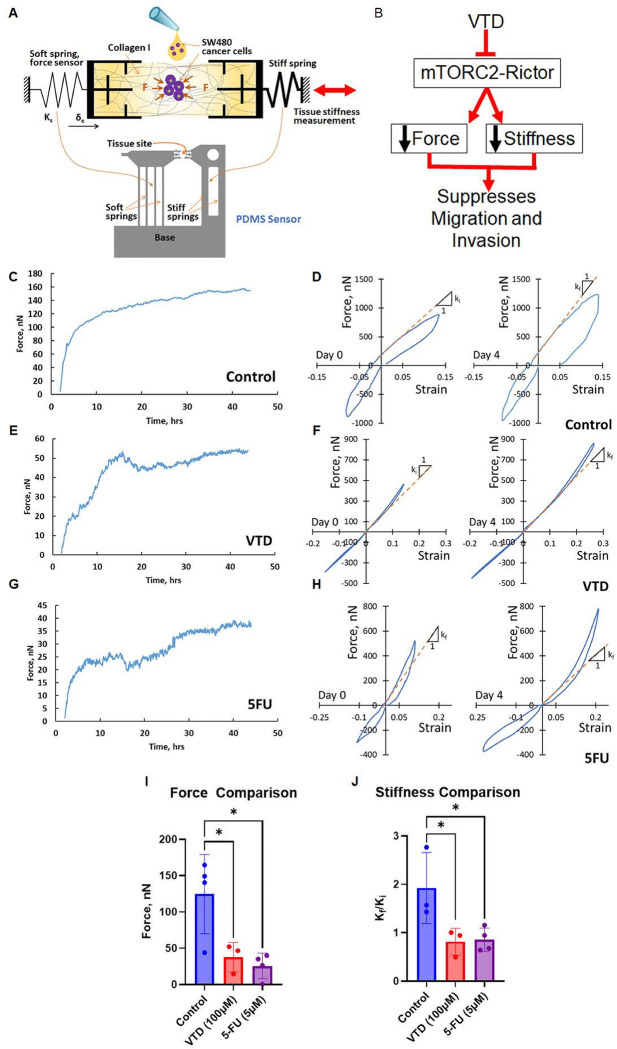
VTD treatment of SW480 cancer cells reduces contractile force and matrix stiffening. Schematic illustration of the self-assembly of tissue specimens and measurement of cell force and tissue stiffness for mechanobiological analysis (**A**). A a droplet of cell-collagen mixture is added between two grips and subsequently polymerized. Cell-generated force (F) is determined from the product of spring constant (K_s_) and deformation (δ_c_). The stiffness of the tissue is measured by applying compression or tension while continuously monitoring force and strain. The design of a PDMS sensor shows the setup’s structure. 5-FU (5μM) was used as a positive anti-cancer control drug. Tissue force dynamics and force-strain curves (Day 0 and Day 4) were measured in control (**C-D**), VTD (100μM) (**E-F**), and 5-FU (5μM) (**G-H**) treated cells. A comparison of 24-hour force shows that both VTD and 5-FU significantly reduced total force compared to DMSO treated cells (**I**). Collagen remodeling in DMSO treated cells results in tissue stiffening in 4 days while VTD and 5-FU treatment result in softening of the tissues (**J**). Day 0 and Day 4 stiffness are referred to as K_i_ and K_f,_ respectively. K_f_/K_i_ indicates the fold change in tissue stiffness. These findings strongly indicate that VTD treatment in SW480 cancer cells can suppress migration and invasion through mTORC2-Rictor-dependent force inhibition and reduce ECM stiffness (**B**). (n=3, *P<0.05, mean ± SD).

To investigate whether VTD can potentially suppress cancer cell migration and invasion by inhibiting cell contractility, we utilized a novel biomechanical sensor (22) that longitudinally measures cellular force and remodeling of 3D matrices. The basic sensor configuration comprises three main components: a soft spring (spring constant, K_s_), a stiff spring, and two grips connected to the springs (Fig. 7A). The soft spring is the sensor that measures force from the cells in the tissue formed between the grips. To construct the specimen, a matrix precursor solution (e.g., collagen or Matrigel matrix) is mixed with cells and dispensed onto the grips, where the tissue is allowed to self-assemble *in situ*. Spring deformation (δ_c_) is measured from brightfield images, and force is quantified as the product of spring constant and deformation (F = K_s_* δ_c_). The stiffness of the tissue was also measured to determine matrix remodeling by the cells. We hypothesized that inhibition of the Rictor-mTORC2-AKT signaling pathway by VTD (100μM) decreases matrix force and stiffness, two key players in cancer cell migration and invasion (Fig. 7B).

SW480 cancer cells were pretreated with VTD (100μM) for 48 hours and 5-FU (5μM) for 24 hours before mixing with collagen to construct tissue samples on the sensors. Cells continued treatment over the duration of the experiment. Force evolution revealed that the tissues reached a steady state within 24 hours (Fig. 7C, E, and G). Alongside quantification of stiffness, video recording revealed this mechanism by showing the interaction between SW480 cell migration and remodeling of the matrix. VTD (100μM) treated cells showed considerable impairment by slower cell migration and a smaller deformation between the sensors, which is attributed to less force (Video S2) compared to DMSO (Video S1). We found that both VTD (100μM) and 5-FU (5μM) significantly inhibited cancer cell contractility and remodeling of the collagen matrix (Fig. 7D, F, and H). High contractile forces in DMSO (control) samples resulted in a ~ 2-fold increase in tissue stiffness. Therefore, VTD and 5-FU decreased force contractility (Fig. 7I) and significantly inhibited matrix stiffening (Fig. 7J). These results indicate that VTD can prevent cancer progression by simultaneously targeting biochemical and biomechanical signaling (69–72). By reducing force and stiffness, VTD can potentially inhibit the force-dependent metastatic crosstalk between cancer and stromal cells, interfering with physicochemical signaling in the tumor microenvironment.

### Veratridine suppresses tumor progression in an AOM/DSS mouse colon cancer model in a UBXN2A-dependent manner

**Figure 8:**
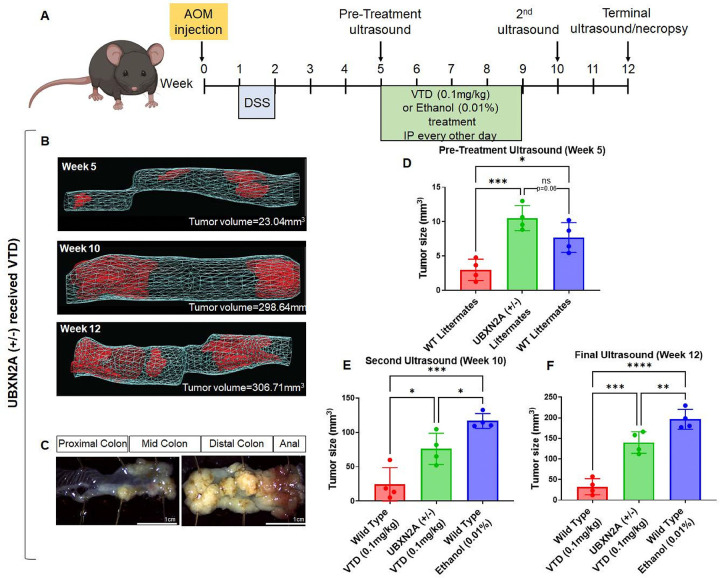
VTD reduces tumor growth in a murine mouse model of CRC in a UBXN2A-dependent manner. Mice received AOM/DSS to generate tumors in both the descending colon and rectum to mimic human CRC (**A**). Progressing tumors were monitored by ultrasound before and after ethanol (0.01%, Control) or VTD treatment (0.1mg/kg) and before necropsy (**A-B**). The tumor growth rate was insignificant between UBXN2A (+/−) (**D**, green) and WT (**D**, blue) at the pre-treatment ultrasound stage due to having half the expression level of UBXN2A. However, the heterogeneous UBXN2A (+/−) mice after receiving VTD (**E-F**, green) had slower tumor growth rates at the second and final ultrasound weeks compared to the WT mice that received ethanol (**E-F**, blue) despite having full UBXN2A expression. WT mice had slower tumor growth rates and smaller tumors (**E-F**, red). Experiments were conducted under the approved IACUC animal protocol in both male and female C57BL/6 mice. (n=5, *P<0.05, **p<0.01, ***P<0.001, ****P<0.0001, mean ± SD).

Based on the above results, we wanted to further examine the effects of VTD on tumor progression using an azoxymethane/dextran sodium sulfate (AOM/DSS) induced colon cancer mouse model. This method of inducing colon cancer tumors has been shown to closely mimic human colorectal cancer by developing tumors in the lower section of the colon and rectum (73). Additionally, the AOM CRC model resembles human CRC at the molecular level in many respects, including the dysregulated AKT/mTORC pathway (74, 75), which is the dominant target of the VTD-UBXN2A therapeutic approach. Following the AOM/DSS protocol, we administered a single injection of AOM in all C57BL/6 mice, followed by a week of DSS in their water. We could detect tumors at week 5 during the pre-treatment ultrasound. After this ultrasound, the mice (4 per group, mixed males and females) received intraperitoneal (IP) injections of ethanol (0.1%, control) or VTD (0.1 mg/kg), the maximum tolerable dose (19), every other day for 28 days (Fig. 8A). There was a total of 14 injections. The timeline and dosage are an optimized VTD treatment regimen with no neuro- or cardiotoxicity (19). Intraluminal gel ultrasound allowed us to quantify cancer progression in live animals by measuring tumor volume by 3D reconstruction of the tumors at weeks 5 (pre-treatment), 10 (2 weeks post-treatment), and 12 (endpoint), as previously described by our group (76). Normal adjacent tissues and colorectal tumors were collected at endpoint from the dissected colon and rectum. The total tumor growth from ethanol (0.01%) treated Wild Type (WT) mice (Fig. S17A-D), VTD (0.1 mg/kg) treated heterozygous haploinsufficient UBXN2A (+/−) mice (Fig. 8B–C), and VTD (0.1 mg/kg) treated WT mice (Fig. S17E-H), was measured at the pre-treatment, post-treatment, and terminal stages. The tumor growth rate was insignificant between UBXN2A (+/−) and WT at the pre-treatment ultrasound stage (Fig. 8D). WT mice treated with VTD (0.1 mg/kg) (Fig. 8E–F, red, and Fig. S17) had significantly slower tumor progression in compared to the ethanol (0.01%, control) group (Fig. 8E–F, blue, and Fig. S17). More importantly, VTD (0.1 mg/kg) decreased tumor growth in the UBXN2A (+/−) mice (Fig. 8E–F, green) despite having half the amount of UBXN2A as their WT counterparts. The inhibited tumor growth observed in weeks 10 and 12 in VTD (0.1 mg/kg) treated UBXN2A (+/−) mice shows that elevation of UBXN2A by VTD can overcome pre-existing low UBXN2A levels and actively slow tumor growth. These results indicate that VTD’s ability to inhibit tumor growth is primarily mediated via the UBXN2A-tumor suppressor axis, whose activation is associated with the suppression of multi-tumorigenic pathways in CRC (10, 12, 14).

## Discussion

This study explored the anti-cancer mechanisms and therapeutic efficacy of a mTORC2 specific potent anti-cancer molecule, VTD, as a plant alkaloid-based UBXN2A enhancer. Although many different organic compounds in plants have medical applications, plant alkaloids, in particular, have led to some of the most important drug developments of the past 50 years (77). Vinca alkaloids, indole alkaloids such as irinotecan, and diterpene alkaloids such as paclitaxel, have become staples in chemotherapy regimens for a wide range of cancers (78).

The role of VTD as a transcriptional inducer of UBXN2A was initially discovered using a high-throughput drug screen. This study utilizes *in vitro* and *in vivo* approaches to reveal that VTD-dependent induction of UBXN2A can target the Rictor-mTORC2 pathway in human colon cancer. Reduction of Rictor, a key member of the mTORC2 pathway, interrupts full activation of the mTORC2-AKT tumorigenic pathway in cancer cells. Examining downstream protein targets and pathways of Rictor-mTORC2 revealed that VTD reduces phosphorylation of Ser^473^ of AKT, negatively controls the population of colon cancer stem cells, slows angiogenesis, and increases both intrinsic and extrinsic apoptosis pathways. Finally, our live cell migration and invasion method, as well as evaluating the mechanobiological response in CRC cells, revealed that VTD can significantly decrease cancer cell migration and invasion in both primary and metastatic colon cancer cells.

It has been shown that pharmacological activation of PTEN, a potent tumor suppressor gene, can dominantly antagonize the proto-oncogenic phosphoinositide 3-kinase (PI3K)–AKT signaling pathway with acceptable side effects (79). However, activation of PTEN functions as a prime antagonist *upstream* of the PI3K-AKT signaling pathway that can dominantly shut down the PI3K-AKT pathway in normal and cancer cells. Furthermore, studies have shown that inhibiting AKT Ser^473^ phosphorylation in the absence of Rictor reduces AKT downstream activity by about 50%, indicating that AKT Ser^473^ phosphorylation by Rictor is only necessary to promote maximal AKT activity (80, 81). The absence of AKT Ser^473^ phosphorylation due to inhibited Rictor can potentially overcome drug resistance and substantially reduce reported side effects associated with complete inhibition of the AKT pathway by PTEN-based therapies. Furthermore, while a mutation in PTEN is near significant in CRC patients, including early-onset, both Rictor and UBXN2A stay wild-type in CRC, according to TumorPortal. The absence of mutations in UBXN2A and Rictor genes increases the potential therapeutic efficacy of VTD as a UBXN2A enhancer and a potent Rictor suppressor in patients with CRC in early- and late-onset as well as the metastatic stage of CRC.

Finally, this study examined the use of VTD *in vivo* and confirmed its role as an anti-tumor agent in an AOM/DSS-induced colon cancer mouse model. Experiments in UBXN2A haploinsufficient mice revealed that the enrichment of UBXN2A by VTD in a time-dependent manner slows the tumor formation in the lower colon and rectum. The response of UBXN2A haploinsufficient mice to VTD, which closely mimics the ~ 50% CRC patients with low UBXN2A levels (10), supporting the drug-likeness of VTD as a potent inhibitor of Rictor-mTORC2 tumorigenic pathways in CRC.

## Materials and methods

The experimental details of “Materials and methods” are included in supplementary materials.

## Data Availability

Our manuscript includes all relevant raw data and will be freely available to any researcher wishing to use it for future non-commercial studies.
